# Biodegradation of Chlorsulfuron and Metsulfuron-Methyl by *Aspergillus niger*

**DOI:** 10.1100/tsw.2002.281

**Published:** 2002-05-18

**Authors:** Elisabetta Zanardini, Marco Negri, Giovanna Boschin, Alessandra D'Agostina, Anna Valle, Anna Arnoldi, Claudia Sorlini

**Affiliations:** Dipartimento di Scienze e Tecnologie Alimentari e Microbiologiche, Sezione MAAE, Università di Milano, via Celoria 2, I-20133 Milano, Italy; Dipartimento di Scienze Molecolari Agroalimentari (DISMA), Sezione di Chimica, Università di Milano, via Celoria 2, I-20133 Milano, Italy

**Keywords:** sulfonylurea herbicides, Aspergillus niger, biodegradation

## Abstract

In this work, investigations were performed under laboratory conditions of the degradation ability by a common soil fungus, *Aspergillus niger*, toward chlorsulfuron and metsulfuron-methyl. The results were very encouraging (79% for chlorsulfuron and 61% for metsulfuron-methyl of total degradation), especially compared to those registered in our previous studies with a Pseudomonas fluorescens strain B2 (about 21 to 32%). Furthermore, the chemical degradation of the two compounds was studied and two products (1*[2-methoxy-benzene-1-sulfonyl]-7-acetyltriuret* and 1*[2-chlorobenzene-1-sulfonyl]-7-acetyltriuret)* were isolated and characterised by hydrolysis in acidic conditions. Our aim in the future will be the identification of intermediate metabolites by HPLC and LC-MS analyses in order to identify the degradative pathway by the fungal strain and to compare this to those obtained by chemical degradation and by *P. fluorescens strain*.

## INTRODUCTION

Sulfonylureas are a relatively new class of products characterised by several aspects that make them particularly interesting compared to other herbicides. Their main characteristics are low field rates (2 to 100 g/ha), high herbicidal activity, broad action spectrum, good crop selectivity, and low human and animal toxicity (DL50 on rat generally >5000 mg/kg). These compounds are used to control broad-leaved weeds in soybean, cereals, rice, corn, tomato, and potato fields.

Their chemical structure is characterised by three portions: an arylic group ($R_1$), a sulfonylurea bridge, and an heterocyclic portion ($R_2$), as shown in Fig. 1. Their mode of action is highly specific on protein synthesis, since these herbicides inhibit acetolactate synthase (ALS), an enzyme of the biosynthetic pathway of the branched amino acids (valine, isoleucine, and leucine) in plant and micro-organisms, but it is not present in animals[1,2].

Sulfonylureas are not very volatile, although some can present a high mobility in soil; the soil adsorption depends on the pH values and on organic matter. These herbicides can be degraded mainly by chemical or microbial processes, while the photodegradation is not relevant[3,4,5,6]. In acidic conditions, they can be chemically hydrolysed via sulfonylura bridge cleavage[4,7,8].

The general aim of this work is to study the biodegradation of two sulfonylureas: chlorsulfuron and metsulfuron-methyl (Figs. 2A and 2B), by a common soil fungus, *Aspergillus niger*. These compounds were chosen because of their high persistence in soil in certain conditions that can cause problems to successive cultures[1]. In previous works[9], the biodegradation of chlorsulfuron and metsulfuron-methyl by a *Pseudomonas fluorescens* strain (named B2), isolated from soil treated with sulfonylurea herbicides, was studied in different laboratory conditions. Low degradation percentages (around 10 to 15%) were obtained after 1 month by adding the compounds as sole carbon and energy sources to a minimal mineral medium. More relevant removal occurred in cometabolic conditions, demonstrating that the enzymatic attack on these molecules by a *P. fluorescens* strain can occur mainly in rich medium. In fact, in Plate Count Broth medium, degradations of 21 and 32% were recorded for metsulfuron-methyl and for chlorsulfuron, respectively, after 2 weeks of incubation. The aim of this work was to test the degradative ability of *A. niger* towards chlorsulfuron and metsulfuron-methyl and to compare the results with those obtained by using the *P. fluorescens* strain B2.

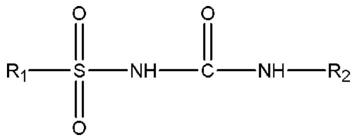

**FIGURE 1.** Chemical structure of sulfonylurea herbicides.

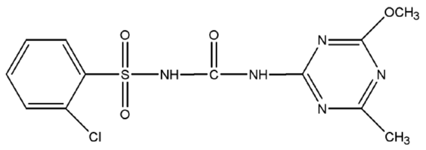

**FIGURE 2A**

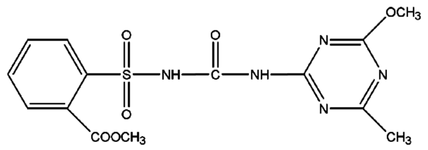

**FIGURE 2B**

**FIGURE 2**. (A) Chlorsulfuron; (B) metsulfuron-methyl.

## EXPERIMENTAL METHODS

### Microbiological Methods

Cometabolic degradation tests were performed in two different media: M9 mineral medium[10] added with 0.2% sodium acetate, and Plate Count Broth (PCB-Difco). *A. niger* was inoculated adding 1 ml of spore suspension of a standard turbidity ($10^6$ spores/ml) to 100 ml of cultural media. Chlorsulfuron (analytical grade 99.5%) and metsulfuron-methyl (analytical grade 97.4%) were kindly provided by DuPont (Italy) and were added to the cultural tests at the concentration of 500 mg/l.

In all experiments, the liquid media used were buffered at neutral pH (6.5 to 7) and the pH was maintained at a neutral value during the experimental period to avoid chemical degradation by acidic hydrolysis. The experimental tests were performed in aerobic conditions with an incubation temperature of 30°C for a period of 21 days. Samples were submitted to HPLC-UV analyses. In parallel with the degradation tests, biotic and abiotic controls were prepared in order to confirm fungal growth and to monitor the chemical degradation.

### HPLC Analyses

Solvents and reagents were used without any purification. Samples for HPLC were filtered through disposable nylon 66 filters (0.45 µm, Alltech) and analysed by HPLC-VWD. HPLC analyses were conducted on a HP-1050 quaternary pump fitted with a Rheodyne injector (20 µl, loop) and equipped with a HP-1050 Variable Wavelength Detector (HPLC-VWD). The system was controlled by HP Chemstations (DOS Series, Hewlett-Packard). Chromatograms were recorded at 254 nm. The column was a Lichrospher® 100 RP-18 (5 µm, 250 ∞ 4 mm, Merck, Darmastad, Germany), the flow rate was 1 ml/min, and the gradient from 20:80 methanol/water +1% acetic acid to 100:0 methanol/water +1% acetic acid over 50 min, then 5 min isocratic.

### Hydrolysis of Chlorsulfuron and Metsulfuron-Methyl in Acidic Conditions

To study the chemical hydrolysis of chlorsulfuron and metsulfuron methyl, 200 mg of each herbicide was dissolved in 20 ml of a mixture 1:1 methanol/water and the pH value of the reaction mixture was adjusted to 1 by adding 1 ml of HCl 6N. The reaction was maintained under stirring for 5 days at room temperature. The mixture was evaporated under vacuum at 60°C and the reaction products were purified by flash-chromatography on silica gel using as eluent 9:1 dichloromethane/methanol. The products obtained were characterised by $^1$H-NMR (Bruker AMX-300; solvent $CDCl_3$).

## RESULTS AND DISCUSSION

### Biodegradation in Cometabolic Condition

In the M9 medium with 0.2% sodium acetate, no significant degradation of either compound was observed, probably because the medium was insufficiently rich to support growth of *A. niger* and the degradation activity. On the other hand, in the PCB medium the biodegradation percentages, after 21 days (excluding chemical degradation), were about 79% for chlorsulfuron and 61% for metsulfuron-methyl (Figs. 3 and 4).

### Chemical Degradation

The complete hydrolysis of chlorsulfuron and metsulfuron-methyl solutions (1000 mg/l) was obtained after only 4 days at a very low pH value (1), under stirring at room temperature. The hydrolysis performed in acidic condition permitted the isolation and the characterisation of two products, different from those obtained by microbial degradation.

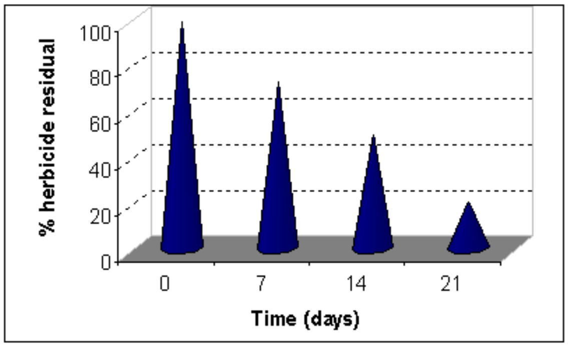

**FIGURE 3**. Biodegradation in PCB of chlorsulfuron (500 mg/l) by *A. niger* strain.

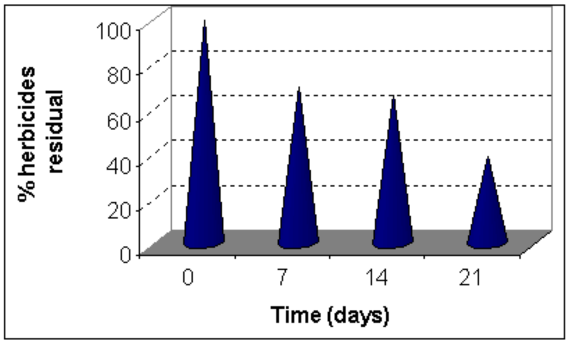

**FIGURE 4.** Biodegradation in PCB of metsulfuron-methyl (500 mg/l) by *A. niger* strain.

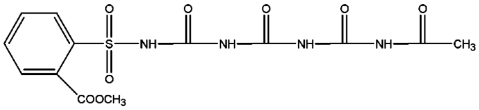

**FIGURE 5**. 1[2-methoxy-benzene-1-sulfonyl]-7-acetyltriuret.

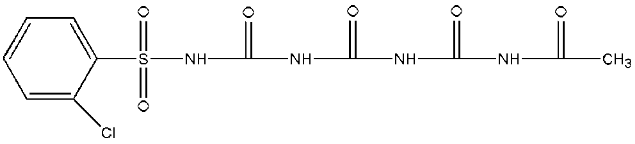

**FIGURE 6.** 1[2-chlorobenzene-1-sulfonyl]-7-acetyltriuret.

The compounds were identified as: 1[2X-benzene-1-sulfonyl]-7-acetyltriuret, where X is Cl for chlorsulfuron, and $COOCH_3$ for metsulfuron-methyl, respectively (Figs. 5 and 6).

## CONCLUSIONS

The biodegradation of the two herbicides operated by *A. niger* preferentially occurs under cometabolic conditions in the presence of a rich medium. In fact, no significant removal was observed in the mineral medium to which 0.2% sodium acetate was added, while relevant degradation percentages were evidenced after 21 days with PCB (79% for chlorsulfuron and 61% for metsulfuron-methyl).

An explanation of this result could be that in the cometabolic conditions the enzymatic attack on chemicals often occurs in no specific way by constitutive enzymes, which are produced in greater quantities when the microbial biomass is abundant. Few authors have reported studies with pure cultures: Joshi and coauthors observed the degradation of chlorsulfuron by a soil actinomycetes, *Streptomyces griseolus*, and two fungal strains, *A. niger* and *Penicillium* sp., in cometabolic conditions[11]. These authors obtained a degradation of 60% in 48 h by *S. griseolus* and a higher degradation by *A. niger* and *Penicillium* sp. in the presence of 75 mg/l of herbicide[11].

More recently, some authors have shown that under laboratory conditions, the microbial degradation observed with pure cultures of *A. niger* was a chemical degradation induced by a decrease of the pH value due to the production of citric acid[12,13]. In our conditions, however, the pH value was monitored during the degradation experiments and remained constant at neutrality which leads us to exclude any chemical degradation. Our future investigations will be focused on the identification of the intermediate metabolites by HPLC and LC-MS analyses in order to identify the degradative pathway by the fungal strain and to compare this to those obtained with the *Pseudomonas fluorescens* B2 strain.

## ACKNOWLEDGEMENTS

This paper was presented at the CSIC/ ESF workshop Analysis, Toxicity and Biodegradation of Organic Pollutants in Groundwater from Contaminated Land, Landfills and Sediments, Barcelona, Spain, 8–10 November, 2001. We are indebted to Dr. Bonacini (DuPont- Italy) for chlorsulfuron and metsulfuron-methyl compounds.
